# Preoperative membranous urethral length predicts quality-of-life recovery and urinary incontinence after holmium enucleation of the prostate

**DOI:** 10.1007/s00345-026-06673-x

**Published:** 2026-08-10

**Authors:** Naoya Nagasaki, Takuya Sadahira, Ichiro Tsuboi, Tomofumi Watanabe, Shin Kanemoto, Yusuke Tominaga, Satoshi Katayama, Shingo Nishimura, Kensuke Bekku, Masami Watanabe, Toyohiko Watanabe, Motoo Araki

**Affiliations:** 1 Department of Urology, Dentistry and Pharmaceutical Sciences, Okayama University Graduate School of Medicine, 2-5-1 Shikata-cho, Kita-ku, Okayama, 700-8558 Japan; 2 Center for Innovative Clinical Medicine, Okayama University Hospital, Okayama, Japan; 3 Department of Urology, Kochi Health Sciences Center, Kochi, Japan; 4 Department of Interdisciplinary Science and Engineering in Health Systems, Okayama University, Okayama, Japan

**Keywords:** Holmium laser enucleation of the prostate, Membranous urethral length, Magnetic resonance imaging, Stress urinary incontinence, Quality of life, Benign prostatic hyperplasia

## Abstract

**Purpose:**

Holmium laser enucleation of the prostate (HoLEP) relieves bladder outlet obstruction, but postoperative quality-of-life (QOL) recovery and stress urinary incontinence (SUI) differ among patients. We evaluated whether preoperative membranous urethral length (MUL) on MRI predicts QOL recovery and SUI after HoLEP, independent of clinical and surgical factors.

**Methods:**

This single-center retrospective study included 135 patients with preoperative MRI. MUL was measured on sagittal T2-weighted images. The primary outcome was 6-month International Prostate Symptom Score (IPSS) -QOL ≤ 1. SUI was defined as the use of ≥1 pad per day at 6 months. IPSS-QOL changes were analyzed with generalized estimating equations. For 6-month QOL recovery and SUI, logistic regression was adjusted for age, enucleated weight, prostate volume, baseline QOL, and surgeon. We evaluated thickness of posterior wall of membranous urethral sphincter (TPWMUS), membranous urethral volume (MUV), and a short-MUL/thin-TPWMUS phenotype.

**Results:**

The 6-month IPSS-QOL ≤ 1 rate was 40.0%; SUI occurred in 19/135 patients (14.1%). Longer MUL was linked to better QOL recovery at every time point (p ≤ 0.015) and independently predicted both 6-month QOL recovery (adjusted odds ratio [aOR] 1.35, 95% CI 1.14–1.61) and lower SUI risk (odds ratio [OR] 0.76, 0.61–0.93), remaining significant after adjustment. Adding MUL improved prediction (AUC for SUI 0.588→0.704; for QOL 0.716→0.760). Across MUL tertiles, QOL recovery rose from 13.9% to 56.1% ,and SUI fell from 20.0% to 2.2%. Short MUL/thin-TPWMUS showed higher SUI and poorer QOL recovery.

**Conclusions:**

Preoperative MUL on MRI independently predicted QOL recovery and continence after HoLEP.

**Supplementary Information:**

The online version contains supplementary material available at 10.1007/s00345-026-06673-x.

## Introduction

Holmium laser enucleation of the prostate (HoLEP) is an established size-independent treatment for benign prostatic obstruction [1, 2]. While objective flow parameters improve reliably, postoperative quality-of-life (QOL) recovery and stress urinary incontinence (SUI) differ among patients. For patients, leakage and urgency matter more than flow measurements.

Previous studies have identified age, body mass index (BMI), prostate volume, urinary retention, and enucleated weight as SUI risk factors after HoLEP [3–6]. Yang et al. reported in a meta-analysis that SUI decreases over time, with prostate volume, age, and BMI as significant predictors [3]. Lee et al. showed in a prospective registry that both stress and urgency urinary incontinence (UI) continuously decreased by 6 months [4]. In addition, magnetic resonance imaging (MRI)-based periurethral anatomy has been examined as a predictor: Fan et al. identified thickness of the posterior wall of the membranous urethral sphincter (TPWMUS), prostatic apex configuration, and membranous urethral volume (MUV) as predictors of SUI after HoLEP [7], and Lee et al. found that each additional millimeter of membranous urethral length (MUL) reduced SUI odds at 1, 3, and 6 months in 147 consecutive patients [8]. Beyond incontinence, preoperative and perioperative factors have also been linked to overall functional outcomes after HoLEP [9].

MUL, the distance from the prostatic apex to the penile bulb, approximates the functional length of the external urethral sphincter, which is most developed around the membranous urethra [10]. Coakley et al. first showed that longer preoperative MUL on MRI predicts earlier continence recovery [11], a relationship since confirmed by meta-analysis [12, 13], and combined sphincter dimensions provide additional information [14]. MUL has also been linked to continence after HoLEP [8, 15, 16]. However, these studies measured leakage alone at isolated visits, not the QOL recovery that matters most to patients. We therefore asked whether MUL predicts QOL recovery and how it changes over time. We evaluated preoperative MUL on MRI as the primary anatomical predictor of longitudinal QOL recovery and SUI after HoLEP, and tested whether it remained significant after adjustment for clinical and surgical factors. We secondarily explored TPWMUS, MUV, and a composite short-MUL/thin-TPWMUS phenotype.

## Materials and methods

### Study design

Retrospective observational study at a single institution, Kochi Health Sciences Center (January 2020 to October 2025). Ethical approval was granted by the Institutional Review Board of Okayama University, which served as the central review board for the study group (approval no. 2606-028). The requirement for individual informed consent was waived given the retrospective design, and patients were informed via an opt-out notification. Patients with neurogenic lower urinary tract symptoms (LUTS) or prior prostate/urethral surgery were excluded. Preoperative pelvic MRI was not routinely performed but was obtained mainly for prostate cancer evaluation; measurements were available in 135 of 469 eligible patients (28.8%). Accordingly, the MRI cohort had higher PSA than the non-MRI cohort (4.82 vs. 1.70 ng/mL). Most procedures (112/135, 83.0%) were performed by a single experienced surgeon (> 300 HoLEP cases).

### Surgical procedure

HoLEP was performed with a holmium laser and tissue morcellator using a two- or three-lobe technique, consistent with our institutional standard [17]. A three-way Foley catheter was removed 2 to 3 days postoperatively. Postoperative evaluation was performed at 1, 3, 6, and 12 months.

### MRI acquisition

MRI was performed at our institution on 1.5- or 3.0-T scanners using a standard pelvic protocol with T2-weighted (turbo spin-echo) sequences in the axial, sagittal, and coronal planes. Measurements were made by a single trained observer blinded to outcomes.

### MRI measurements (Fig. 1A)

MUL was measured on the midsagittal T2-weighted image, along the course of the urethra, from the prostatic apex to the entry of the urethra into the penile bulb [7, 12]; these landmarks correspond to those recommended for standardized MUL measurement [18]. TPWMUS was measured on axial T2 images as the maximum posterior wall thickness of the membranous urethral sphincter at maximum cross-sectional width [7]. UWT was the maximum wall thickness on the same images. MUV = π × MUL × (UWT/2)²/1000 (cm³), analyzed per 0.1 cm³ [7].

**Fig. 1:**
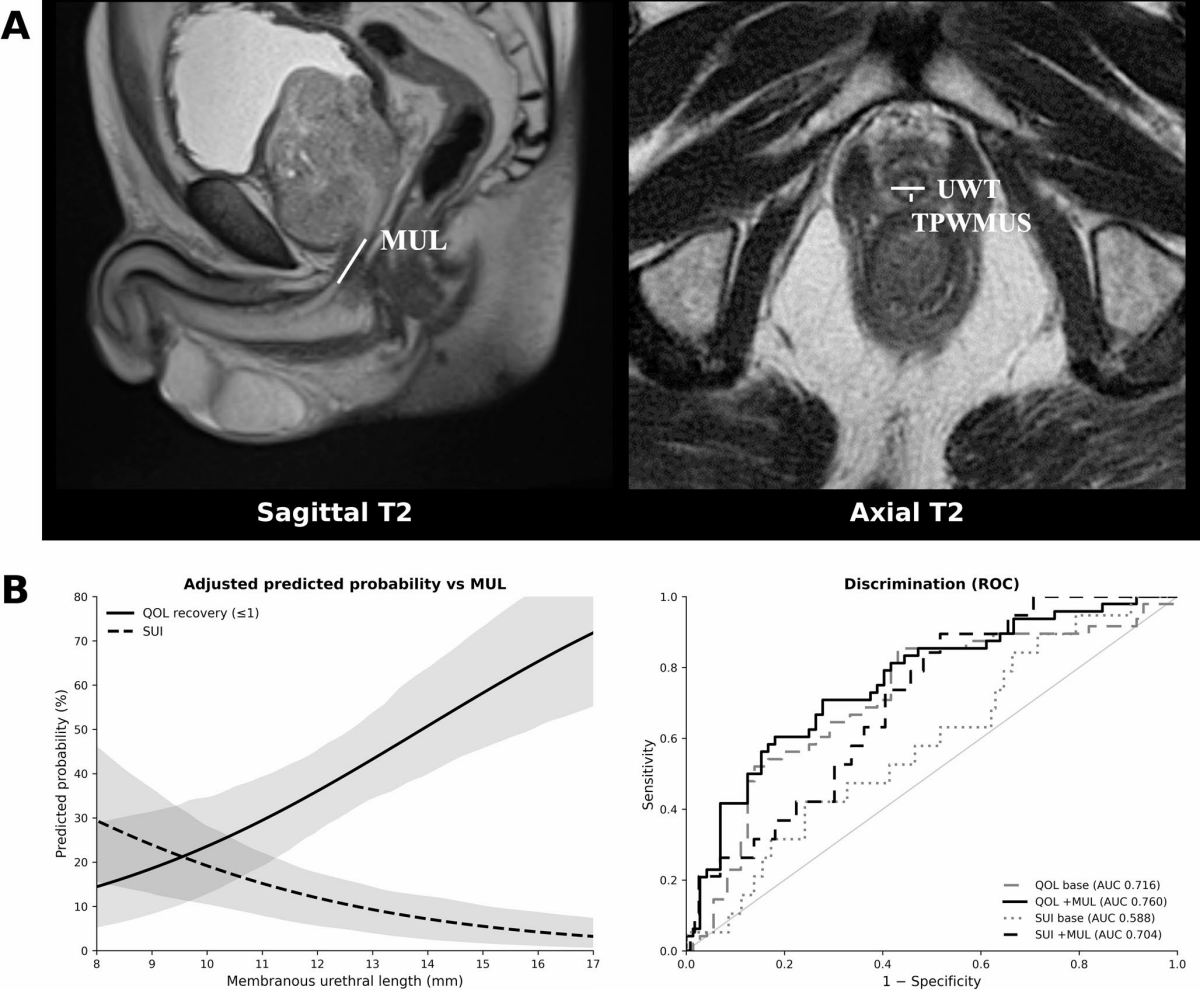
Preoperative membranous urethral length (MUL) and postoperative outcomes after HoLEP. **A** Representative MRI measurements. Left: MUL on a mid-sagittal T2-weighted image, measured from the prostatic apex to the bulbar urethra. Right: thickness of the posterior wall of the membranous urethral sphincter (TPWMUS) and urethral wall thickness (UWT) on an axial T2-weighted image at the membranous urethra. **B** Predicted probabilities and discrimination as a function of preoperative MUL. **Left**: adjusted predicted probability of 6-month IPSS-QOL recovery and stress urinary incontinence (SUI), with 95% confidence bands, with age and enucleated weight held at their means. **Right**: receiver operating characteristic (ROC) curves for the same outcomes, comparing the clinical base model (age + enucleated weight) with the MUL-based model. AUC, area under the curve

### Outcomes

The primary outcome was 6-month IPSS-QOL ≤ 1, the global patient-reported bother item (≤ 1 indicating a satisfied state). IPSS (overall symptom severity) and SUI (incontinence) were analyzed separately to distinguish obstruction relief from sphincteric continence, which reflect different anatomy. SUI was defined as the use of ≥ 1 pad per day at 6 months. Secondary outcomes were longitudinal IPSS and IPSS-QOL changes at 1, 3, 6, and 12 months, and SUI at 6 months.

### Statistical analysis

Longitudinal IPSS-QOL changes (424 person-time observations) were analyzed using generalized estimating equations (GEE) with an exchangeable working correlation structure and robust standard errors (Stata 19.5 xtgee), adjusted for baseline IPSS-QOL, age per 10 years, enucleated weight per 10 g, MUL, time, and MUL-by-time interaction (ρ = 0.493). Logistic regression was used for binary outcomes; odds ratios (ORs) with 95% confidence intervals (CIs) are reported. Both univariable and multivariable analyses are reported for all candidate variables. MUL was analyzed as a continuous variable and also categorized into tertiles for dose-response evaluation. TPWMUS, MUV, and a short-MUL/thin-TPWMUS group (both MUL and TPWMUS below the cohort medians: MUL < 12.01 mm and TPWMUS < 2.86 mm) were analyzed as exploratory measures. Each MRI parameter was added separately to the clinical base (age + enucleated weight) model, and discrimination was assessed by the area under the ROC curve (AUC) with 95% CIs. For the primary MUL models, we checked for confounding by adding prostate volume, baseline IPSS-QOL, and operating surgeon in a fully adjusted model. Calibration was evaluated by the Hosmer–Lemeshow test and calibration slope, overall accuracy by the Brier score, and optimism-corrected AUC by bootstrap resampling (500 replicates). Binary outcomes used complete cases; the GEE model used all observations. All multivariable models contained 3 predictors (EPV: SUI 6.3, QOL 16.0). Analyses used Stata 19.5 (StataCorp, College Station, TX, USA); two-sided *p* < 0.05 was significant.

## Results

### Patients and characteristics

After excluding 13 patients with neurogenic LUTS or prior prostate/urethral surgery, 135 of 469 eligible patients had preoperative MRI and formed the study cohort. The median IPSS was 18, IPSS-QOL 5, and the mean MUL 12.2 mm (Table 1). Compared with the 334 eligible patients who did not undergo preoperative MRI, the study cohort had a higher median PSA (4.82 vs. 1.70 ng/mL), whereas preoperative symptom burden was similar (Supplementary Table 1).

**Table 1:** Characteristics of the patients included in the study (*N* = 135)

	Mean ± SD or median (IQR)
Age (years)	71.9 ± 8.4
BMI (kg/m²)	23.9 ± 3.1
Acute urinary retention history	24/135 (17.8%)
Long-term urethral catheterization	12/135 (8.9%)
IPSS total	18 (12–24)
IPSS-QOL	5 (4–5)
PVR (mL)	39.4 (11.8–72.8)
PSA (ng/mL)	4.8 (1.9–8.1)
Prostate volume (mL)	51.3 ± 27.5
MUL (mm)	12.16 ± 2.84
TPWMUS (mm)	2.93 ± 0.90
UWT (mm)	10.40 ± 1.59
MUV (cm³)	1.08 ± 0.47
Operative time (min)	109 (90–135)
Enucleated weight (g)	16.0 (9.0–35.0)
Catheter duration (day)	3.6 ± 2.3
Hospital stay (day)	6.9 ± 2.2

MUL, membranous urethral length; TPWMUS, thickness of posterior wall of membranous urethral sphincter; UWT, urethral wall thickness; MUV, membranous urethral volume (= π × MUL × (UWT/2)²/1000 cm³); IPSS, International Prostate Symptom Score; QOL, quality of life; PVR, post-void residual; PSA, prostate-specific antigen. IPSS, IPSS-QOL, PVR, PSA, enucleated weight, and operative time are presented as median (IQR) owing to skewed distributions; all other continuous values are mean ± SD

IPSS improvement was substantial (median ΔIPSS 7 at 6 months, 8 at 12 months), consistent with effective obstruction relief. The 6- and 12-month IPSS-QOL ≤ 1 rates were 40.0% and 38.7% (Table 2). At 6 months, SUI was present in 19/135 patients (14.1%) (Table 3).

**Table 2:** Postoperative functional outcomes at each time point

Follow-up time	ΔIPSS, median (IQR)	ΔQOL, median (IQR)	IPSS-QOL ≤ 1	QOL improvement ≥ 2
1 month	2 (− 2 to 10)	1 (0–2)	19/120 (15.8%)	49/119 (41.2%)
3 months	5 (− 1 to 13)	1 (0–3)	26/99 (26.3%)	46/98 (46.9%)
6 months	7 (2–14)	2 (0–4)	48/120 (40.0%)	69/117 (59.0%)
12 months	8 (2–15)	2 (1–3)	36/93 (38.7%)	55/90 (61.1%)

ΔIPSS and ΔQOL = preoperative minus postoperative (positive value = improvement)

**Table 3:** Univariable and multivariable logistic regression for SUI and 6-month IPSS-QOL ≤ 1

	Univariable OR (95% CI)	*p*	Multivariable OR (95% CI)	*p*
A. 6-month SUI (*n* = 135; events = 19; EPV = 6.3 per variable)				
Age (per 10 years)	1.53 (0.83–2.83)	0.173	—	
Enucleated weight (per 10 g)	1.01 (0.81–1.26)	0.934	—	
MUL (per 1 mm)	0.75 (0.61–0.92)	0.006	0.76 (0.61–0.93)	0.008
TPWMUS (per 1 mm)	0.53 (0.29–0.97)	0.040	0.54 (0.29–0.99)	0.047
MUV (per 0.1 cm³)	0.89 (0.79–1.01)	0.066	0.90 (0.79–1.02)	0.102
UWT (per 1 mm)	0.93 (0.69–1.26)	0.645	0.98 (0.71–1.35)	0.884
B. 6-month IPSS-QOL ≤ 1 (*n* = 120; events = 48; EPV = 16.0 per variable)				
Age (per 10 years)	0.91 (0.59–1.42)	0.687	—	
Enucleated weight (per 10 g)	1.32 (1.09–1.59)	0.004	—	
MUL (per 1 mm)	1.38 (1.17–1.63)	< 0.001	1.35 (1.14–1.61)	< 0.001
TPWMUS (per 1 mm)	1.13 (0.76–1.69)	0.548	1.25 (0.82–1.93)	0.302
MUV (per 0.1 cm³)	1.19 (1.08–1.30)	< 0.001	1.17 (1.05–1.29)	0.003
UWT (per 1 mm)	1.33 (1.03–1.71)	0.026	1.23 (0.94–1.61)	0.132

Multivariable OR: adjusted for age and enucleated weight (— = not entered in model); base AUC: SUI = 0.588, QOL = 0.716. For 6-month QOL analysis, TPWMUS was available in 119 patients (events = 47). For MUL-augmented models: AUC (95% CI) 0.704 (0.593–0.815) for SUI and 0.760 (0.672–0.848) for QOL; optimism-corrected AUC 0.671 and 0.740; Brier 0.113 and 0.194; Hosmer–Lemeshow *p* = 0.71 and 0.79

### Longitudinal QOL and regression analyses

Longer MUL was associated with greater IPSS-QOL improvement at every time point, with the association strengthening over time (β, the change in IPSS-QOL per 1-mm increase in MUL, ranged from + 0.120 at 1 month to + 0.187 at 12 months; all *p* ≤ 0.015). MUL independently predicted both 6-month QOL recovery (adjusted odds ratio [aOR] 1.35, 95% CI 1.14–1.61, *p* < 0.001) and 6-month SUI (odds ratio [OR] 0.76, 0.61–0.93, *p* = 0.008) (Table 3). MUL remained significant after adding prostate volume, baseline IPSS-QOL, and operating surgeon in the fully adjusted model (QOL aOR 1.25, 1.04–1.50; SUI aOR 0.65, 0.50–0.84). MUL did not predict the degree of total IPSS improvement (*p* = 0.54), which depended instead on enucleated weight (*p* < 0.001), whereas SUI was predicted by MUL but not enucleated weight (*p* = 0.63).

### Model performance

The MUL-based models were adequately calibrated (Hosmer–Lemeshow *p* = 0.71 and 0.79; Brier scores 0.113 and 0.194). Adding MUL to the clinical base model (age + enucleated weight) increased discrimination (Fig. 1B): for SUI, the AUC rose from 0.588 to 0.704 (95% CI 0.593–0.815), and for 6-month QOL recovery, from 0.716 to 0.760 (0.672–0.848). The corresponding optimism-corrected AUCs were 0.534 and 0.671 for SUI, and 0.697 and 0.740 for QOL recovery. Adding the short-MUL/thin-TPWMUS group to the base model gave AUCs of 0.722 for SUI and 0.737 for 6-month QOL recovery. At 12 months, longer MUL remained associated with QOL recovery (aOR 1.24, 95% CI 1.03–1.49; AUC 0.753) (Supplementary Table 2).

### MUL tertiles and the short-MUL/thin-TPWMUS group

MUL tertiles showed a clear dose-response: 6-month QOL recovery increased from 13.9% in the shortest to 56.1% in the highest tertile (aOR per tertile 2.55, 95% CI 1.53–4.26, *p* < 0.001), while SUI fell from 20.0% to 2.2% (aOR per tertile 0.47, 95% CI 0.23–0.94, *p* = 0.032) (Fig. 1B). Patients with short-MUL/thin-TPWMUS group (23.9%) had higher SUI (31.2% vs. 8.8%; aOR 4.49, 95% CI 1.60–12.54, *p* = 0.004) and lower QOL recovery (24.1% vs. 44.4%; aOR 0.33, 0.12–0.92, *p* = 0.035). Within the short-MUL group (SUI 20.9%, QOL recovery 21.1%), thin TPWMUS further raised SUI (31.2%) but not QOL recovery (24.1%).

## Discussion

In this cohort, a longer preoperative MUL on MRI predicted better patient-reported recovery after HoLEP, with faster QOL recovery, lower SUI, and a tertile gradient; these associations held after adjustment for prostate volume, baseline symptom severity, and operating surgeon.

MUL reflects the functional length of the membranous urethra and its surrounding rhabdosphincter. During HoLEP, apical enucleation can mechanically stress the external urethral sphincter, a recognized cause of transient SUI [7]; a longer preoperative MUL preserves more functional sphincter and may improve tolerance to this injury. A similar relationship holds in radical prostatectomy, where longer MUL predicts earlier continence recovery [12, 13]. The strengthening MUL–QOL association over time (β + 0.120 to + 0.187) fits ongoing sphincteric recovery.

Earlier MRI studies linked periurethral anatomy to post-HoLEP incontinence and focused on early recovery: Oka et al. found that longer MUL predicted earlier continence but saw no difference beyond 6 months [15], while Ueki et al. and Smani et al. reported similar early associations [16, 19]. Most recently, Lee et al. confirmed that MUL predicts stress incontinence at 1–6 months, but, like earlier work, used leakage at single timepoints [8]. Our study differs in three respects: patient-reported QOL recovery is the primary outcome, its trajectory is modeled longitudinally rather than at isolated visits, and the determinants of symptom relief (enucleated weight) are separated from those of continence (MUL). MUL predicted perceived recovery, not just leakage, with effects on QOL and 6-month SUI largely unchanged after adjustment for surgeon and baseline severity. In our data, the degree of symptom (IPSS) improvement was driven by enucleated weight, whereas SUI related to MUL; both enucleated weight and MUL were associated with QOL recovery. Postoperative urinary incontinence is itself a significant determinant of health-related quality of life after prostate surgery [20], which may explain why MUL — through its link to continence and possibly other pathways — was associated with patient-reported QOL recovery in this cohort.

Exploratory cross-sectional measures were less consistent. TPWMUS was associated with SUI but not QOL recovery, opposite to Fan et al. [7].; given the few SUI events, this requires prospective confirmation. MUV is calculated from MUL and urethral diameter, so its link with QOL recovery most likely reflects MUL rather than urethral volume itself. The short-MUL/thin-TPWMUS group reflected mainly short MUL: within the short-MUL group, thin TPWMUS further raised SUI but not QOL recovery. With data-derived cut-offs and few events, we treated it as exploratory only.

Continence recovery after HoLEP reflects both baseline sphincter anatomy and operative handling of the apex. Contemporary continence-preserving modifications, including anteroposterior dissection, en-bloc enucleation with early apical release, anterior prostatic urethral mucosal flap preservation, meticulous apical mucosal preservation, and anterior fibromuscular stroma-preserving dissection, aim to limit traction and thermal injury to the external urethral sphincter [21–26]. Reported benefits are not uniform: a multicenter propensity-matched series found no reduction in incontinence with early apical release [27]. Because the use and completeness of these maneuvers were not graded prospectively here, and interrater agreement for apical mucosal preservation is limited even among experts [28], their effects could not be separated from those of MUL. Whether men with a shorter MUL benefit disproportionately from such techniques is hypothesis-generating and requires prospective testing.

The contribution of MUL to discrimination persisted after internal validation. After bootstrap correction for optimism, the AUC rose from 0.534 to 0.671 for SUI and from 0.697 to 0.740 for QOL recovery, corresponding to corrected increments of 0.137 and 0.043. For SUI the corrected increment slightly exceeded the apparent increment, indicating that the gain is not an artefact of overfitting. Age and enucleated weight alone provided limited discrimination for continence, and MUL contributed useful additional information. The absolute level of discrimination remains moderate, as would be expected of a single anatomical variable, and the extent to which it improves clinical decisions is a question for prospective work incorporating calibration and clinical net benefit. In the meantime, MUL offers a readily available preoperative measurement for individualized risk communication and a foundation on which fuller prediction models can be built.

The 6-month SUI rate of 14.1% falls within the published range of 4–15% [3–6, 8, 29]. The 6-month timepoint matches most HoLEP outcome studies and is our most complete follow-up (*n* = 120) [3–6, 8]. Strengths include two complementary patient-centered outcomes, adjustment for surgeon and baseline severity, and bootstrap internal validation.

This study has several limitations. It was a retrospective, single-center analysis with data-derived cutoffs requiring external validation, low EPV (6.3), attrition at 12 months (42/135, 31%), and no validated SUI severity instrument; postoperative SUI was assessed only at 6 months, so the durability of the MUL–continence association could not be tested. Intraobserver and interobserver agreement of the MRI measurements was not assessed. The reported reproducibility of manual MUL measurement varies widely (ICC approximately 0.34–0.89) and is lowest when the plane and landmarks are not standardized [18]; agreement improves with a defined measurement protocol [30] or dedicated reader training [31]. Our landmarks correspond to the recommended convention [18], and the observer was blinded to outcome, so residual measurement error should be non-differential and would attenuate rather than inflate the associations, whereas the data-derived cutoffs are more sensitive to measurement variability. Although operating surgeon was included in the fully adjusted models and the MUL associations persisted, the few procedures performed by other surgeons preclude separating surgeon experience, technique, and case selection; because continence is influenced by the enucleation learning curve and apical handling, results from this high-volume setting may not generalize to lower-volume surgeons or centers. Finally, MRI was obtained for cancer evaluation rather than systematically, and the cohort differed from the 334 eligible patients without MRI (Supplementary Table 1). The two groups were comparable in the characteristics most relevant to the present outcomes. Preoperative IPSS, IPSS-QOL, prostate volume, postvoid residual urine volume, and history of acute urinary retention did not differ. The differences that were observed, namely younger age, smaller enucleated weight, and higher PSA, reflect the indication for imaging rather than the lower urinary tract symptoms under study, so referral for MRI is unlikely to have acted through MUL or through recovery conditional on MUL. Absolute event rates may nonetheless vary with case mix, and confirmation in consecutive multicenter cohorts using standardized imaging would extend these findings to unselected HoLEP populations.

MUL is best regarded as a risk-enrichment marker rather than a decision threshold. Where pelvic MRI is already available, a shorter MUL may prompt more detailed counseling about delayed continence and QOL recovery and encourage particularly meticulous apical and mucosal preservation. It should not be used to exclude patients from HoLEP, nor to justify MRI performed solely for continence prediction.

## Conclusion

Preoperative MUL on MRI was associated with QOL recovery and SUI after HoLEP, and these associations remained after adjustment for clinical and surgical factors. When MRI is already available, MUL can help patients prepare for recovery and make better-informed decisions. Larger prospective studies are needed to confirm its value in HoLEP risk assessment.

## Supplementary Information

Below is the link to the electronic supplementary material.

Supplementary Material 1

## Data Availability

The data that support the findings of this study are available from the corresponding author upon reasonable request.

## Abbreviations

aOR: Adjusted odds ratio
AUC: Area under the curve
BPH: Benign prostatic hyperplasia
CI: Confidence interval
EPV: Events per variable
GEE: Generalized estimating equations
HoLEP: Holmium laser enucleation of the prostate
IPSS: International prostate symptom score
LUTS: Lower urinary tract symptoms
MRI: Magnetic resonance imaging
MUL: Membranous urethral length
MUV: Membranous urethral volume
OR: Odds ratio
PSA: Prostate-specific antigen
PVR: Postvoid residual urine volume
QOL: Quality of life
TPWMUS: Thickness of posterior wall of membranous urethral sphincter
SUI: Stress urinary incontinence
UI: Urinary incontinence
UWT: Urethral wall thickness
