# Supplementary material for: Preoperative membranous urethral length predicts quality-of-life recovery and urinary incontinence after holmium enucleation of the prostate
Source: World J Urol. 2026 Aug 10;44(1):552. doi: 10.1007/s00345-026-06673-x (PMC13457498; doi:10.1007/s00345-026-06673-x)
Supplement: Supplementary file 1 [file 345_2026_6673_MOESM1_ESM.docx]

**Supplementary Table 1. Comparison of eligible patients with and without preoperative MRI.**

|  | **Preoperative MRI (n = 135)** | **No preoperative MRI (n = 334)** | **p value** |
| --- | --- | --- | --- |
| Age (years) | 72.1 ± 8.4 | 75.3 ± 7.6 | <0.001 |
| BMI (kg/m²) | 24.0 ± 3.1 | 23.6 ± 3.7 | 0.35 |
| PSA (ng/mL) | 4.82 (1.9–8.1) | 1.70 (1.0–5.9) | 0.006 |
| Prostate volume (mL) | 43.4 (31.8–67.8) | 40.8 (30.8–69.6) | 0.72 |
| Preoperative PVR (mL) | 39.3 (11.7–70.8) | 49.3 (14.0–72.6) | 0.47 |
| Acute urinary retention history | 23/131 (17.6%) | 16/97 (16.5%) | 0.97 |
| IPSS total | 18 (12–24) | 19 (13–26) | 0.18 |
| IPSS-QOL | 5 (4–5) | 5 (3–6) | 0.95 |
| Enucleated weight (g) | 16.0 (9.0–35.0) | 25.0 (10.2–45.0) | 0.012 |

Eligible patients were those who underwent HoLEP during the study period after exclusion of neurogenic lower urinary tract symptoms and prior prostate or urethral surgery. Values are mean ± SD, median (interquartile range), or n/N (%), following the conventions of Table 1. Groups were compared with the unpaired t test (age, BMI), the Mann–Whitney U test (PSA, prostate volume, PVR, IPSS total, IPSS-QOL, enucleated weight), or the chi-square test (acute urinary retention history). BMI, body mass index; HoLEP, holmium laser enucleation of the prostate; IPSS, International Prostate Symptom Score; MRI, magnetic resonance imaging; PSA, prostate-specific antigen; PVR, postvoid residual urine volume; QOL, quality of life; SD, standard deviation.

**Supplementary Table 2. Univariable and multivariable logistic regression for 12-month IPSS-QOL recovery.**

|  | **Univariable OR (95% CI)** | ***p*** | **Multivariable OR (95% CI)** | ***p*** |
| --- | --- | --- | --- | --- |
| **12-month IPSS-QOL ≤ 1 (n = 93; events = 36)** | | | | |
| Age (per 10 years) | 0.69 (0.42–1.14) | 0.150 | — | — |
| Enucleated weight (per 10 g) | 1.37 (1.08–1.75) | 0.011 | — | — |
| MUL (per 1 mm) | 1.31 (1.10–1.55) | 0.002 | 1.24 (1.03–1.49) | 0.021 |

Multivariable model adjusted for age and enucleated weight (— = not entered). MUL-based model AUC 0.753 (base model 0.741). QOL recovery across MUL tertiles: 19.4%, 41.9%, and 54.8%. Postoperative urinary incontinence was assessed only at 6 months and was not available as a separate 12-month measure. MUL, membranous urethral length; QOL, quality of life; OR, odds ratio; CI, confidence interval; AUC, area under the curve.

**Supplementary Figure 1. Time course of patient-reported quality-of-life outcomes after HoLEP.**

The proportion of patients reaching a satisfied state (IPSS-QOL ≤ 1) and the proportion with meaningful QOL improvement (≥ 2 points) are shown at 1, 3, 6, and 12 months. Both improved progressively, with the satisfied-state rate plateauing near 6 months and QOL improvement continuing to rise through 12 months. IPSS-QOL, International Prostate Symptom Score quality-of-life item.
